# Assessing D-dimer test specificity for pulmonary embolism diagnosis in emergency departments

**DOI:** 10.1016/j.clinsp.2026.101037

**Published:** 2026-07-09

**Authors:** Zohair Al Aseri, Faisal Owaid, Yasser Aloufi, Fares Altowariqi, Abdulaziz Almehbash, Younis Alsulami, Saleh Alrawdhan, Khalid Alqahtani, Ayman El-Faham

**Affiliations:** a Department of Clinical Medical Sciences, College of Medicine, Dar Al Uloom University, Saudi Arabia; b Departments of Emergency Medicine and critical care, College of Medicine, King Saud University, Riyadh, Saudi Arabia; c Adult Critical Care, Therapeutic Deputyship, Ministry of Health, Riyadh, Saudi Arabia

**Keywords:** Pulmonary embolism, D-dimer, Emergency department, Diagnostic accuracy, CTPA

## Abstract

Pulmonary Embolism (PE) is a potentially life-threatening condition that requires rapid and accurate diagnosis in emergency settings. Although Computed Tomography Pulmonary Angiography (CTPA) is the reference standard, its routine use is limited by cost, radiation exposure, and contrast-related risks. This study aimed to evaluate the diagnostic performance of D-dimer testing in patients suspected of PE. A retrospective cross-sectional study was conducted at King Khalid University Hospital, Riyadh, Saudi Arabia, including 1,316 patients who underwent D-dimer testing between January and December 2023. CTPA was used as the reference standard in patients selected for imaging based on clinical assessment. The prevalence of confirmed PE was 8.4%. D-dimer demonstrated high sensitivity (87.4%) and excellent negative predictive value (97.4%), supporting its role as a reliable rule-out tool. However, specificity was low (43.9%) and the positive predictive value was limited (12.5%), reflecting a high rate of false-positive results. Application of the age-adjusted D-dimer threshold reclassified 23 patients, resulting in a modest improvement in specificity from 43.9% to 45.8%, while maintaining diagnostic safety. Receiver operating characteristic analysis showed modest discriminative ability (AUC = 0.656). D-dimer levels were significantly associated with increasing age and male sex, while nationality showed no significant effect. In conclusion, D-dimer testing is an effective initial screening tool for excluding PE in emergency departments; however, its low specificity limits its standalone diagnostic value. Integration with clinical prediction rules and age-adjusted thresholds may improve diagnostic accuracy and reduce unnecessary imaging.

## Highlights

• D-dimer testing demonstrates high sensitivity and an excellent negative predictive value for ruling out pulmonary embolism in emergency departments.
• The test shows limited specificity and low positive predictive value, leading to a high false-positive rate.
• Increased age and male gender are associated with significantly higher D-dimer levels.
• Reliance on D-dimer testing can help reduce unnecessary CTPA imaging when used as an initial screening tool.
• D-dimer remains a valuable, cost-effective strategy for excluding pulmonary embolism in acute care settings.

## Background

Pulmonary Embolism (PE) is a potentially fatal cardiovascular emergency that occurs when athrombus, most commonly originating from the deep veins of the lower extremities, obstructs the pulmonary arterial circulation.^1^ Venous thromboembolism, which includes both deep vein thrombosis and pulmonary embolism, represents a major public health burden. In the United States alone, it is estimated to affect up to 900,000 individuals annually.^2^ If left untreated, PE can lead to severe complications and significant mortality, highlighting the importance of timely identification and appropriate management.^3^ However, therapeutic interventions such as anticoagulation therapy may also carry risks, particularly bleeding complications, making an accurate diagnosis essential before initiating treatment.^4^

Several diagnostic modalities are currently used to evaluate suspected pulmonary embolism. Computed Tomography Pulmonary Angiography (CTPA) has become the most widely used imaging modality and is considered the reference standard for confirming the diagnosis due to its high diagnostic accuracy.^5^ Despite its effectiveness, CTPA is associated with certain limitations, including radiation exposure, potential adverse reactions to contrast agents, and the requirement for specialized radiological resources.^6^ For this reason, clinical prediction tools such as the Wells score and the Geneva score are commonly used to estimate the pre-test probability of PE and guide further diagnostic testing.^7^ Additional supportive diagnostic methods include ventilation-perfusion scans, venous compression ultrasonography, end-tidal carbon dioxide measurement (PetCO_2_), and laboratory biomarkers.^8^

Among laboratory investigations, D-dimer has gained considerable importance in the diagnostic workup of suspected thromboembolic disease. D-dimer is a fibrin degradation product generated during fibrinolysis when cross-linked fibrin is broken down by plasmin.^9^ Elevated levels may indicate the presence of active clot formation and degradation; however, the marker is not specific to thromboembolic disease because various physiological and pathological conditions, such as liver disease, trauma, pregnancy, malignancy, inflammation, and advanced age, may also increase D-dimer levels.^10^^,^^11^

Despite this limitation, D-dimer testing is widely used due to its high sensitivity in detecting thrombotic conditions, making it particularly valuable for excluding pulmonary embolism in patients with a low or intermediate clinical probability.^12^^,^^13^ In individuals with low pre-test probability and D-dimer levels below the conventional threshold (commonly 500 µg/L), the likelihood of PE is sufficiently low that further imaging can often be avoided.^13^

Unnecessary imaging in hospitals and emergency departments is increasing the risks of health hazards to both patients and health practitioners, and by using the D-dimer test, these can be reduced or even avoided. Moreover, the D-dimer test can be provided within minutes, helping patients reduce the time of stay in the hospital and increase the capacity and efficiency in the emergency department. Also, the D-dimer test is considered “cost – effective” unlike advanced imaging, and this is crucial in terms of economic value to both government and private hospitals.

Under physiological conditions, D-dimer levels are typically undetectable in healthy individuals unless coagulation and fibrinolysis have been activated.^14^ The test is typically performed using immunoassay-based techniques such as Enzyme-Linked Immunosorbent Assay (ELISA) or immunoturbidimetric methods that detect specific epitopes on the D-dimer fragment.^15^

Nevertheless, the diagnostic value of D-dimer is affected by its relatively low specificity, which may lead to a high number of false-positive results and unnecessary imaging investigations.^16^, ^17^, ^18^ To address this limitation, several diagnostic strategies have been proposed, including age-adjusted D-dimer thresholds. This approach adjusts the cutoff value for patients older than 50-years (age × 10 µg/L), improving specificity while maintaining acceptable sensitivity.^19^^,^^20^ Evidence from multiple meta-analyses supports the safety and effectiveness of this strategy in excluding PE among patients with low or intermediate pre-test probability.^21^

Although numerous international studies have evaluated the diagnostic performance of D-dimer testing in suspected pulmonary embolism, limited research has investigated its diagnostic characteristics, particularly specificity, within the Saudi Arabian population.^22^ D-dimer cut-off value is largely validated in areas of North America and Europe and is influenced by regional comorbidities. Validating this value in the studied region will help local health practitioners to accurately estimate the risk and benefit of this crucial test. Also, the regional population, especially in Saudi Arabia, is younger than in North America and Europe, and this can affect the standard cut-off value of D-dimer, and the present research would help in assessing the accuracy and validity specific to the studied region. Furthermore, recent clinical algorithms integrating age-adjusted D-dimer thresholds with clinical probability assessments, such as the YEARS algorithm, have demonstrated promising results in safely reducing unnecessary imaging in emergency department settings.^23^

Therefore, this study aims to evaluate the diagnostic performance of the D-dimer test in patients with suspected pulmonary embolism and to examine its role in clinical decision-making within an emergency care setting. Specifically, the study assesses the ability of D-dimer testing to exclude pulmonary embolism in patients presenting with symptoms such as dyspnea and chest pain, using Computed Tomography Pulmonary Angiography (CTPA) as the confirmatory diagnostic standard.

## Material and methods

### Study design and setting

This retrospective cross-sectional analytical study was conducted using electronic medical records from the Emergency Department (ED) of King Khalid University Hospital (KKUH), Riyadh, Saudi Arabia. The study period extended from January 1 to December 31, 2023. The study aimed to evaluate the diagnostic performance of D-dimer testing for the detection of Pulmonary Embolism (PE) in patients presenting to the ED suspected of thromboembolic disease.

Patient identification was initially performed by retrieving all adult patients who underwent D-dimer testing during the study period. D-dimer testing was ordered as part of the routine diagnostic evaluation for patients presenting with clinical features suggestive of pulmonary embolism, such as shortness of breath, chest pain, or unexplained hypoxia, according to the treating physician’s clinical assessment.

Computed Tomography Pulmonary Angiography (CTPA) was not performed systematically for all patients. Instead, the decision to proceed with CTPA was made by the treating physician based on the overall clinical evaluation, including symptoms, clinical judgment, and assessment of pre-test probability using standard diagnostic approaches applied in routine emergency department practice.

For the purpose of diagnostic accuracy analysis, patients who subsequently underwent CTPA were identified and included in the final analytical cohort. CTPA served as the reference standard for confirming the diagnosis of pulmonary embolism. Radiology reports were retrieved from the hospital radiology information system, and the diagnosis of PE was determined based on the official radiologist's interpretation documented in the patient's medical record.

Information was systematically extracted from electronic medical records using a structured data collection form to ensure consistency and accuracy of the collected data. From an initial pool of 1360 patients who underwent D-dimer testing during the study period, 44 patients were excluded based on predefined criteria, resulting in a final study sample of 1316 participants.

Age-adjusted D-dimer thresholds were applied to patients older than 50-years to account for the physiological increase in D-dimer levels with age. The adjusted cutoff was calculated using the formula: age/100 (mg/L). In the present study’s sample of 1,360 patients, 575 were aged above 50-years and eligible for age-adjusted analysis; of these, 535 were included, while 40 were excluded based on the predefined inclusion and exclusion criteria.

### Inclusion and exclusion criteria

Adult patients aged 18-years or older who presented to the emergency department and underwent D-dimer testing during the study period were eligible for screening. For the diagnostic accuracy analysis, patients who had both D-dimer test results and CTPA imaging available were included, as CTPA was used as the reference standard for confirming pulmonary embolism.

Plasma D-dimer levels were measured in the hospital laboratory using an immunoturbidimetric assay on the STA-Liatest D-dimer analyzer (Diagnostica Stago, France). The results were reported in Fibrinogen Equivalent Units (FEU), and the conventional diagnostic cutoff value of 0.5 µg/mL FEU was used for the evaluation of suspected pulmonary embolism.

Patients were excluded if they had conditions known to significantly elevate D-dimer levels independent of thromboembolic disease, including active malignancy, recent surgery, pregnancy, or inflammatory disorders, in order to minimize confounding factors affecting D-dimer interpretation. Patients with incomplete or missing medical records that prevented adequate data extraction were also excluded. This study is reported in accordance with the STARD 2015 (Standards for Reporting of Diagnostic Accuracy Studies) guidelines.

### Statistical analysis

Categorical variables were summarized using frequencies and percentages, while continuous variables were described using means with standard deviations, medians, ranges, and interquartile ranges. The diagnostic performance of the D-dimer test at the conventional cutoff value of 0.5 µg/mL was evaluated by comparing D-dimer results with CTPA findings. Sensitivity, specificity, Positive Predictive Value (PPV), and Negative Predictive Value (NPV) were calculated along with their corresponding 95% Confidence Intervals.

Comparisons of mean D-dimer levels between demographic and diagnostic groups were performed using independent samples t-tests for variables with two categories and one-way analysis of variance (ANOVA) for variables with more than two groups. The association between patient age and D-dimer levels was examined using linear regression analysis.

Receiver Operating Characteristic (ROC) curve analysis was performed to evaluate the overall diagnostic performance of D-dimer in predicting pulmonary embolism. Statistical significance was defined as a p-value < 0.05. All statistical analyses were performed using IBM SPSS Statistics version 29.0.

For comparison, the conventional fixed cutoff value of 0.5 mg/L was also applied. The diagnostic performance of both approaches was evaluated by comparing their classification outcomes, including the proportion of patients reclassified when using the age-adjusted method.

Age-adjusted D-dimer thresholds were applied for patients older than 50-years to account for the physiological increase in D-dimer levels with age. The adjusted cutoff value was calculated using the formula: age/100 (mg/L). Patients were then classified as positive or negative based on this age-adjusted threshold.

The study received ethical approval from the Institutional Review Boards of Dar Al Uloom University (DAU) and King Khalid University Hospital (KKUH). Patient confidentiality was maintained by anonymizing all data prior to analysis, and no identifiable personal information was included in the dataset.

## Results

**Fig. 1:**
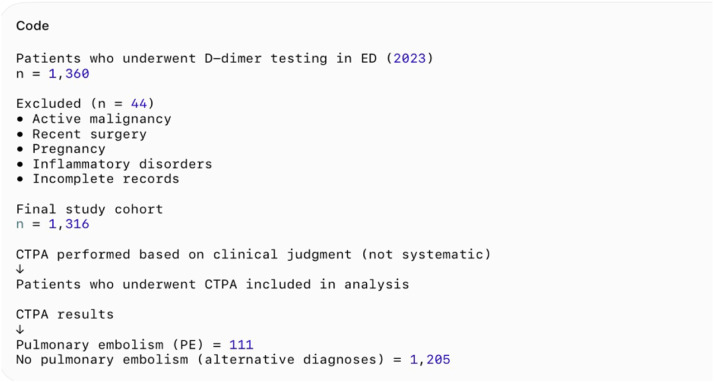
Flow diagram of patient selection according to STARD 2015 guidelines.

Out of 1360 patients initially screened, 44 were excluded based on inclusion and exclusion criteria; 1316 participants met the inclusion criteria (Fig. 1).

Females represented the majority, 806 (61.2%), compared to males, 510 (38.8%). The age distribution was nearly even between young adults aged 18–39 years, 524 (39.8%), and middle-aged adults 40–64 years, 523 (39.7%), while older adults over 65-years comprised 269 (20.4%). The mean age was 47.6 years (SD = 18.2), which ranged from 18- to 109-years. Most were Saudi nationals , 1,127 (85.6%), while 189 (14.4%) were non-Saudi.

Descriptive analysis shows the mean D-dimer was 2.12 (SD = 3.64), with a median of 0.65, IQR 0.33–2.07, and values ranging from 0.01 to 19.77. Overall, 543 participants (41.3%) had a negative D-dimer result, whereas 773 (58.7%) tested positive (Table 1).

**Table 1:** Sociodemographic and other parameters of participants (n = 1316).

		Frequency, n (%)
**Gender**	Female	806 (61.2)
	Male	510 (38.8)
**Age**	Young Adults (18‒39 years)	524 (39.8)
	Middle-Aged Adults (40‒64 years)	523 (39.7)
	Older Adults (> 65 years)	269 (20.4)
	Mean (SD)	47.6 (18.2)
	Range	18‒109
**Nationality**	Non-Saudi	189 (14.4)
	Saudi	1127 (85.6)
**D-Dimer Status**	Negative	543 (41.3)
	Positive	773 (58.7)
		**µg/mL**
**Descriptives statistics of d-Dimer**	Mean (SD)	2.12 (3.64)
	Median	0.65
	Range	0.01 to 19.77
	IQR	0.33 to 2.07

(n) Frequency, (%) Percentages.

**Fig. 2:**
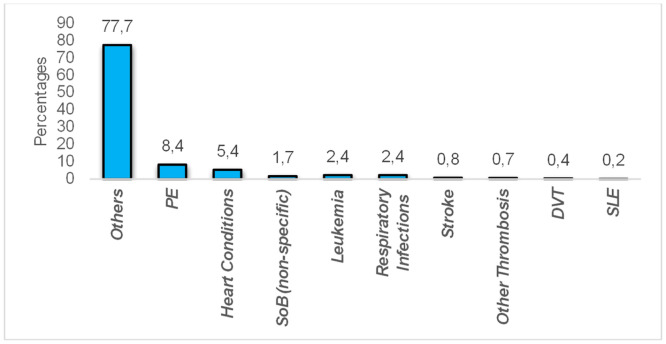
Distribution of different categories of final diagnosis (n = 1316).

Fig. 2 shows the distribution of final diagnostic categories among the study cohort (n = 1316). Most patients were classified under the “Others” group (77.7%). Pulmonary embolism accounted for 8.4% of diagnoses, followed by heart conditions (5.4%). Less frequent diagnoses included shortness of breath of non-specific cause (1.7%), leukemia (2.4%), respiratory infections (2.4%), stroke (0.8%), and other thrombosis (0.7%). Deep venous thrombosis (0.4%) and systemic lupus erythematosus (0.2%) were the least common categories.

Table 2 represented the diagnostic performance of D-dimer at the 0.5 µg/mL cut-off compared with CTPA findings. Among the 111 patients with CTPA-confirmed PE, 97 (87.4%) had elevated D-dimer values (True Positive), while a smaller group 14 (12.6%) were missed despite having PE (False Negative). In contrast, among 1,205 patients without PE, more than half , 676 (56.1%), still showed a positive D-dimer (False Positive), leaving only 529 (43.9%) correctly classified as negative (True Negative). The relationship between D-dimer positivity and CTPA findings was statistically significant (p < 0.001).

**Table 2:** Diagnostic accuracy of D-dimer at the 0.5 µg/mL cut-off compared with CTPA findings.

		D-Dimer (Cut off = 0.5)		Total	Sig. values
		Positive	Negative		
**Pulmonary Embolism**	Yes	97 (87.4%) (TP)	14 (12.6%) (FN)	111 (100.0%)	<0.001
	No	676 (56.1%) (FP)	529 (43.9%) (TN)	1205 (100.0%)	

Table 3 represented the detailed diagnostic performance indices of D-dimer at the 0.5 µg/mL cut-off in excluding pulmonary embolism compared with CTPA. The test demonstrated high sensitivity, correctly identifying 97 of 111 patients with PE (87.4%), while specificity was relatively low, with only 529 of 1205 patients without PE correctly classified as negative (43.9%). The negative predictive value was excellent, with 529 of 543 patients (97.4%) showing a negative D-dimer truly free of PE, confirming its reliability as a rule-out test. These findings indirectly suggest that D-dimer testing was predominantly applied in a population with low to intermediate clinical probability, consistent with its recommended use in routine clinical practice. In contrast, the positive predictive value was poor, with only 97 of 773 patients (12.5%) with a positive D-dimer having PE, reflecting a high false-positive rate.

**Table 3:** Diagnostic performance indices of D-dimer at the 0.5 µg/mL cut-off in excluding pulmonary embolism compared with CTPA.

	**n/Total**	**%**
**Sensitivity**	97 / 111	87.4%
**Specificity**	529 / 1205	43.9%
**PPV**	97 / 773	12.5%
**NPV**	529 / 543	97.4%

To evaluate the potential impact of the excluded patients, an exploratory sensitivity analysis was performed. The 44 excluded patients, who demonstrated significantly higher D-dimer levels, would likely have been classified as D-dimer positive; therefore, they were considered additional false-positive results. Accordingly, the estimated specificity would decrease slightly from 43.9% to approximately 42.4%, and the positive predictive value from 12.5% to about 11.9%. This suggests that the exclusion criteria may have resulted in a modest overestimation of specificity; however, the overall diagnostic performance remains largely unchanged (Table 4).

**Table 4:** Diagnostic performance of conventional versus age-Adjusted D-dimer cutoffs.

	Conventional Cutoff	Age-Adjusted Cutoff
**Sensitivity**	87.4%	87.4%
**Specificity**	43.9%	45.8%
**PPV**	12.5%	12.9%
**NPV**	97.4%	97.5%

After applying the age-adjusted D-dimer threshold, 23 patients were reclassified from positive to negative. All reclassified patients were confirmed to have no pulmonary embolism on CTPA, indicating that no false negatives were introduced. This resulted in an improvement in specificity from 43.9% to 45.8%, while sensitivity remained unchanged at 87.4%. The positive predictive value showed a slight increase, and the negative predictive value remained high. These findings support the safety and effectiveness of the age-adjusted strategy in reducing false-positive results without compromising diagnostic accuracy.

**Fig. 3:**
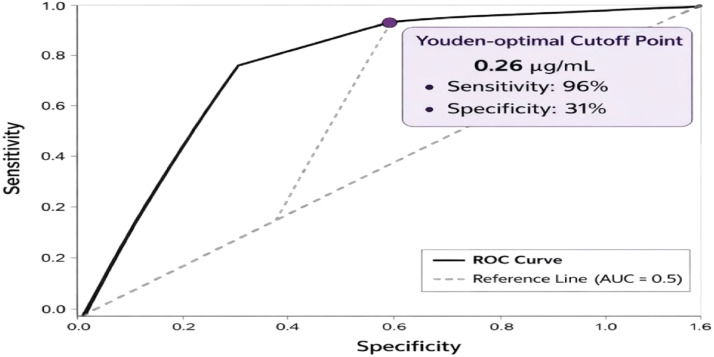
ROC curve analysis demonstrated an AUC of 0.656 (95% CI 0.610–0.703, p < 0.001).

Fig. 3 receiver operating characteristic (ROC) curve illustrating the diagnostic performance of D-dimer for predicting pulmonary embolism using CTPA as the reference standard. The Area Under the Curve (AUC) was 0.656 (95% CI 0.610–0.703, p < 0.001), indicating modest discriminative ability. The conventional cutoff value of 0.5 µg/mL yielded a sensitivity of 87.4% and specificity of 43.9%. The optimal cutoff determined using the Youden index was approximately 0.26 µg/mL, which improved sensitivity to 96% but reduced specificity to 31%, demonstrating the trade-off between sensitivity and specificity in this cohort.

**Fig. 4:**
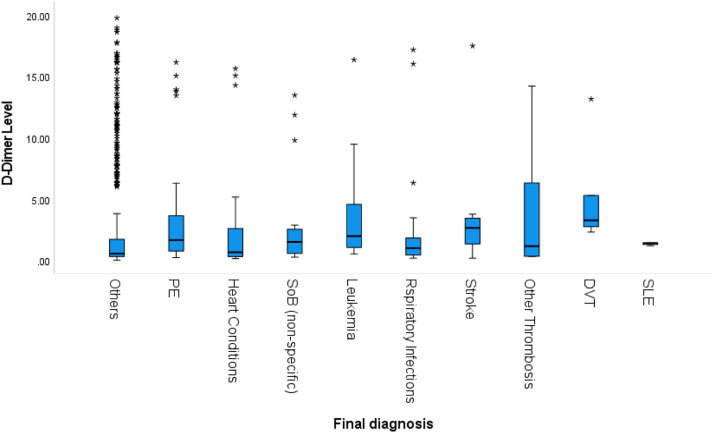
Boxplot showing comparison of mean D-dimer levels across diagnostic subgroups.

Table 5 illustrates the comparison of mean D-dimer levels across demographic and diagnostic subgroups. Males had significantly higher mean D-dimer levels than females (2.84 vs. 1.67 µg/mL, p < 0.001). Age also showed a significant association, with older adults (> 65-years) having the highest mean level (3.35 µg/mL), followed by middle-aged (2.08 µg/mL) and young adults (1.55 µg/mL) (p < 0.001). Nationality did not significantly influence D-dimer values (p = 0.354). Excluded cases had higher mean levels compared to included participants (3.02 vs. 2.07 µg/mL, p = 0.038). Across diagnoses, DVT patients had the highest mean D-dimer (5.35 µg/mL), followed by other thrombosis (3.77 µg/mL) and stroke (3.70 µg/mL). The lowest values were seen in systemic lupus erythematosus (1.32 µg/mL). Differences across diagnostic categories were statistically significant (p = 0.001) as shown in Fig. 4.

**Table 5:** Comparison of mean D-dimer levels across demographic and diagnostic subgroups.

		Mean (SD)	95% CI (Lower–Upper)	*t*/F Value	p-value
**Gender**	Female	1.67 (2.86)	1.49 – 1.88	5.94	**<0.001^a^**
	Male	2.84 (4.15)	2.50 – 3.21		
**Age**	Young Adults	1.55 (2.92)	1.30 – 1.82	23.64	**<0.001^b^**
	Middle-Aged Adults	2.08 (3.45)	1.79 – 2.40		
	Older Adults	3.35 (4.15)	2.83 – 3.87		
**Nationality**	Non-Saudi	2.34 (3.86)	1.80 – 2.90	0.86	0.354**^a^**
	Saudi	2.08 (3.40)	1.89 – 2.29		
**Excluding and Including Status**	Included	2.07 (3.44)	1.89 – 2.28	4.32	**0.038^a^**
	Excluded	3.02 (3.82)	2.12 – 3.98		
**Final Diagnosis**	Others	1.90 (3.35)	1.70 – 2.12	3.02	**0.001^b^**
	PE	2.99 (3.50)	2.37 – 3.74		
	Heart Conditions	2.20 (3.44)	1.44 – 3.08		
	SoB (non-specific)	3.14 (4.06)	1.39 – 5.18		
	Leukemia	3.69 (3.86)	2.42 – 5.28		
	Respiratory Infections	2.49 (4.31)	1.10 – 4.24		
	Stroke	3.70 (4.99)	1.62 – 7.56		
	Other Thrombosis	3.77 (5.13)	0.60 – 7.81		
	DVT	5.35 (4.51)	2.52 – 9.50		
	SLE	1.32 (0.12)	1.19 – 1.39		

(a) Independent samples *t*-test was used for variables with two groups, while (b) One-way ANOVA was used for variables with more than two groups. Values are presented as mean (SD) with 95% Confidence Intervals.

**Fig. 5:**
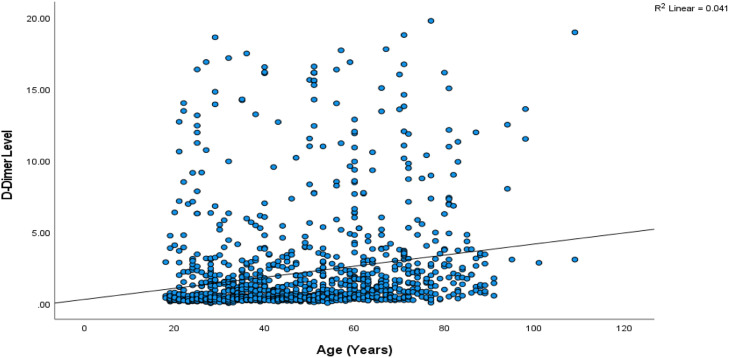
Correlation between Age and D-Dimer level.

Fig. 5 shows the correlation between age and D-dimer levels. A statistically significant positive association was observed, with age explaining approximately 4.1% of the variance in D-dimer values (R^2^ = 0.041, p < 0.001). The regression model indicated that for each additional year of age, the mean D-dimer level increased by 0.039 µg/mL (95% CI 0.028–0.049). The standardized coefficient (β = 0.203, p < 0.001) confirmed that age is an independent predictor of higher D-dimer concentrations. Although the strength of the correlation was modest, the consistent upward trend supports the clinical observation that advancing age is associated with elevated baseline D-dimer levels, which may reduce the specificity of this biomarker in older populations.

Table 6 illustrates the mean D-dimer levels stratified by nationality. Saudi participants had a mean of 2.08 µg/mL (SD = 3.40). Among non-Saudi groups, Yemeni (3.36 µg/mL) and Indian (2.76 µg/mL) participants showed higher mean values, whereas Sudanese (1.67 µg/mL), Filipino (1.41 µg/mL), Syrian (1.04 µg/mL), and Egyptian (0.81 µg/mL) participants demonstrated lower levels. Jordanian participants (2.60 µg/mL) also showed moderately elevated means. Despite numerical differences across subgroups, the overall comparison did not reach statistical significance (p = 0.184), and effect sizes were negligible, suggesting that nationality was not an independent predictor of D-dimer variation in this cohort (Table 7).

**Table 6:** Mean D-dimer levels stratified by nationality (p = 0.184).

	Mean (SD)	95% CI (Lower–Upper)	Effect Size (Bias)
**Saudi**	2.08 (3.40)	1.89 – 2.28	0.0017
**Yemeni**	3.36 (4.50)	1.82 – 5.23	0.0005
**Indian**	2.76 (5.19)	1.04 – 4.86	−0.0168
**Sudanese**	1.67 (2.17)	0.86 – 2.85	−0.0216
**Filipino**	1.41 (2.00)	0.61 – 2.43	−0.0256
**Syrian**	1.04 (1.09)	0.50 – 1.72	0.0129
**Egyptian**	0.81 (0.83)	0.43 – 1.31	0.0027
**Jordanian**	2.60 (4.63)	0.79 – 5.56	−0.0221
**Bangladeshi**	4.07 (6.54)	0.40 – 9.31	−0.0708
**Others**	2.64 (3.70)	1.50 – 3.92	−0.0399

**Table 7:** Bayesian simulation illustrating how changes in pulmonary embolism prevalence influence the predictive values of D-dimer while sensitivity and specificity remain constant.

PE Prevalence	PPV	NPV
5%	7.6%	98.5%
8.4% (study cohort)	12.5%	97.4%
10%	14.8%	96.9%
20%	28.0%	93.3%
30%	40.0%	89.0%

The Bayesian scenario analysis demonstrates that PPV increases substantially with higher disease prevalence, while NPV correspondingly decreases. These findings highlight the strong dependence of predictive values on pre-test probability.

These results highlight that while the D-dimer test is highly sensitive, its specificity is compromised by confounding factors such as age, comorbidities, and non-thrombotic conditions, underscoring the importance of integrating age-adjusted thresholds and clinical scoring systems.

## Discussion

The present study assessed the diagnostic accuracy of D-dimer testing in 1316 emergency department patients evaluated for suspected Pulmonary Embolism (PE). The overall prevalence of confirmed PE was 8.4%, which lies at the lower end of reported rates in international ED cohorts.^16^ Previous studies have shown that diagnostic yields from CT Pulmonary Angiography (CTPA) vary considerably from 8% to 30% depending on case selection and pre-test probability.^10^^,^^12^^,^^13^ As expected in lower-prevalence settings, the Positive Predictive Value (PPV) of D-dimer was reduced, resulting in a high number of false-positive results, a trend that was clearly reflected in this data.^10^^,^^12^^,^^13^

Using the conventional cutoff value of 0.5 µg/mL, the D-dimer test demonstrated relatively high sensitivity (87.4%) and an excellent negative predictive value (97.4%). These findings support the clinical utility of D-dimer as a screening tool in the evaluation of suspected pulmonary embolism. However, the sensitivity observed in the present cohort was slightly lower than the > 95% sensitivity commonly reported in guideline-based diagnostic algorithms. This difference may be related to the retrospective design, patient selection characteristics, and the absence of standardized clinical probability stratification in the present study.

Although formal Pre-Test Probability (PTP) scoring systems such as the Wells or Geneva score were not systematically documented, D-dimer testing in the present institution is typically integrated into a structured clinical workflow. In routine practice, the test is primarily ordered for patients with low to intermediate clinical suspicion of pulmonary embolism, while those with high clinical probability are generally referred directly to imaging without D-dimer testing.

This practice pattern is supported by the observed study findings, particularly the low prevalence of pulmonary embolism (8.4%) and the high negative predictive value (97.4%). Together, these indicators are consistent with the expected performance of D-dimer in low- to intermediate-risk populations, suggesting appropriate clinical utilization despite the absence of formally recorded scoring systems.

Nevertheless, the lack of standardized and extractable PTP documentation remains an important methodological limitation, as it precluded stratified analysis and direct comparison with guideline-based diagnostic algorithms. However, specificity remained low (43.9%), with a correspondingly poor PPV (12.5%).

To further illustrate the influence of disease prevalence on predictive values, a Bayesian scenario analysis was performed assuming constant sensitivity (87.4%) and specificity (43.9%). As expected, the positive predictive value increased substantially with higher disease prevalence, rising from approximately 7.6% at a prevalence of 5% to about 40% at a prevalence of 30%. In contrast, the negative predictive value decreased modestly as prevalence increased.

These findings highlight that the relatively low PPV observed in the present cohort (12.5%) is primarily a function of the low pulmonary embolism prevalence (8.4%) rather than a limitation of the D-dimer test itself. This behavior is consistent with the well-known Bayesian properties of diagnostic tests and reflects the intended clinical role of D-dimer as a high-sensitivity rule-out tool in low to intermediate pre-test probability populations. These findings are consistent with published literature, where D-dimer typically demonstrates sensitivity near 95%–100% but specificity below 50% in unselected ED populations.^10^^,^^12^^,^^13^^,^^16^^,^^23^ Systematic reviews continue to describe D-dimer as a highly sensitive but weakly specific biomarker, particularly in settings with frequent comorbidities such as infection, cancer, trauma, pregnancy, or advanced age, all of which were present in the present cohort.

In the present study, the ROC curve analysis demonstrated a modest discriminative ability of D-dimer for predicting pulmonary embolism (AUC = 0.656). This sensitivity remains below the commonly recommended benchmark of > 95% for safely ruling out pulmonary embolism. When the optimal cutoff was determined using the Youden index, a lower threshold of approximately 0.26 µg/mL was identified, which increased sensitivity to 96% but further reduced specificity. This finding highlights the trade-off between sensitivity and specificity and suggests that adjusting the cutoff value may improve rule-out performance in specific populations, although it may also increase false-positive results and unnecessary imaging. Therefore, clinical decision rules and patient risk stratification should continue to guide the interpretation of D-dimer results in practice. These findings reinforce that D-dimer is well-suited for exclusion of PE but lacks sufficient specificity to confirm PE, especially in high-comorbidity populations.

A strong positive correlation was observed between patient age and D-dimer concentration (Fig. 5), consistent with well-established evidence that D-dimer levels rise physiologically with aging, leading to increased false positives among elderly patients. Clinical trials such as ADJUST-PE have shown that using an age-adjusted cut-off (age × 10 ng/mL in patients ≥ 50-years) significantly improves specificity while maintaining diagnostic safety. In this study, the application of age-adjusted D-dimer thresholds in patients older than 50-years demonstrated improved diagnostic efficiency by reducing false-positive results associated with the conventional 0.5 mg/L cutoff. In this study, the application of the age-adjusted D-dimer threshold led to the reclassification of 23 patients from positive to negative, none of whom had pulmonary embolism on CTPA, confirming the safety of this approach. This strategy resulted in a modest improvement in specificity by reducing false-positive results without affecting sensitivity. Clinically, this may help decrease unnecessary imaging, radiation exposure, and healthcare costs in the emergency setting. These findings are consistent with previous studies, including the ADJUST-PE trial, supporting the use of age-adjusted thresholds as part of an integrated diagnostic approach (Table 4). Although a high proportion of patients remained positive, reflecting the test’s low specificity, these findings support the use of age-adjusted values alongside clinical assessment tools to minimize unnecessary imaging and optimize patient management, particularly within the regional population, where demographic characteristics may influence test performance.

Overall, the results highlight the need for integrated diagnostic pathways that combine D-dimer testing with validated prediction rules (Wells, Geneva, YEARS),^7^^,^^23^ alongside the use of age-adjusted thresholds. Such approaches have demonstrated improved specificity without losing safety and have been shown to reduce unnecessary CTPA utilization in emergency settings.^6^

Given the low incidence of PE and the large proportion of false-positive D-dimer results in the present sample, incorporating age-adjusted thresholds or validated clinical algorithms could help reduce reliance on CTPA and thereby limit radiation exposure, contrast-related risks, financial cost, and ED overcrowding.^16^

Patients with confirmed DVT, stroke, or malignancy exhibited the highest D-dimer levels (Table 5), whereas autoimmune disease (e.g., SLE) cases showed comparatively lower values. This pattern supports the understanding that D-dimer reflects global fibrinolytic activity rather than a specific marker of thromboembolism. Elevated levels are also frequently reported in sepsis, severe inflammation, liver dysfunction, and post-operative states; therefore, D-dimer must be interpreted within the context of clinical probability rather than as a standalone diagnostic indicator.^9^^,^^13^

Although mean D-dimer values were slightly higher in males, no meaningful diagnostic advantage was found, consistent with previous reports showing that age ‒ but not sex ‒ is the primary demographic factor influencing D-dimer interpretation.

One limitation of this study relates to the exclusion of patients with conditions known to elevate D-dimer levels independently of pulmonary embolism, including active malignancy, recent surgery, pregnancy, and inflammatory disorders. The excluded patients demonstrated significantly higher mean D-dimer levels compared with the included cohort. Although these criteria were applied to minimize confounding and improve the interpretability of D-dimer results, their exclusion may have resulted in a modest overestimation of the test’s specificity and positive predictive value. In routine emergency department practice, such patients are frequently encountered, and their inclusion could increase the number of false-positive D-dimer results. Therefore, the reported diagnostic performance may reflect a slightly more selected population than that typically observed in real-world clinical settings, which may limit the generalizability of the findings.

In summary, while the D-dimer test is a valuable tool in the diagnostic evaluation of PE, its limitations regarding specificity and positive predictive value must be acknowledged. An integrated approach that combines D-dimer testing with clinical prediction rules and age-adjusted thresholds may enhance diagnostic accuracy and improve patient outcomes in emergency settings. By tailoring diagnostic strategies to individual patient profiles, healthcare providers can optimize the use of D-dimer testing and reduce the burden of unnecessary imaging and interventions.

## Conclusion

This research confirms that D-dimer testing remains an effective initial screening tool for ruling out pulmonary embolism in emergency care settings. Its high sensitivity and strong negative predictive value make it reliable for excluding PE and reducing unnecessary imaging procedures. However, its low specificity and high false-positive rates significantly limit its use as a standalone diagnostic tool.

These findings highlight the importance of integrating D-dimer testing with clinical assessment tools and applying age-adjusted cutoff values to enhance diagnostic accuracy. Such importance is appreciated in reducing unnecessary imaging modalities in emergency departments due to the increased risks of health hazards to both patients and health practitioners. Furthermore, such tailored approaches can improve the quality of patient care. Since the D-dimer test can be provided within minutes, this will help patients in reducing the time of stay in hospital, increase the capacity and efficiency in the emergency department, optimize the use of healthcare resources, and minimize exposure to unnecessary radiation and contrast agents.

It is important to note that D-dimer cutoff values have been largely validated in North America and Europe and may be influenced by regional comorbidities. Therefore, validating these cutoff values in the studied region would support local healthcare providers in more accurately assessing the risks and benefits of this essential test. Additionally, the relatively younger population in Saudi Arabia compared to Western countries may impact the appropriateness of standard cutoff values, further emphasizing the need for region-specific evaluation, as addressed in this research.

## Authors’ contributions

Conceptualization: Zohair Al Aseri and Ayman El-Faham. Data curation and formal analysis Faisal Owaid, Yasser Aloufi, Fares Altowariqi, Abdulaziz Almehbash, Younis Alsoulmi, Saleh Alrawdhan, Khaled Alqahtan. Investigation: Ayman El-Faham, Zohair Al Aseri. Methodology: Faisal Owaid, Yasser Aloufi, Fares Altowariqi, Abdulaziz Almehbash, Younis Alsulami, Saleh Alrawdhan, Khalid Alqahtani. Supervision Validation: Ayman El-Faham, Zohair Al Aseri. Writing-original draft: all Authors. Writing-review & editing: Ayman El-Faham and Zohair Al Aseri.

## Data availability

The datasets analyzed during the current study were stored at the Emergency Department KKUH. Due to patient confidentiality and ethical restrictions, the data are not publicly available but may be obtained from the corresponding author upon reasonable request and with permission from the institutional ethics committee.

## Ethical statement

This study was conducted in accordance with the ethical standards of the Declaration of Helsinki and approved by the Institutional Review Boards of Dar Al Uloom University (DAU) and King Khalid University Hospital (KKUH). All patient data were anonymized before analysis to ensure confidentiality, and no identifiable information was collected or stored. Due to the retrospective nature of the study and the use of de-identified medical records, the requirement for informed consent was waived by the approving IRBs.

## Funding

This study received no external funding.

## Declaration of competing interest

The authors declare no conflicts of interest.
